# Genetic Algorithm-Based Grouping Strategy for IEEE 802.11ah Networks

**DOI:** 10.3390/s23020862

**Published:** 2023-01-12

**Authors:** Eduard Garcia-Villegas, Alejandro Lopez-Garcia, Elena Lopez-Aguilera

**Affiliations:** 1 Department of Network Engineering, Universitat Politècnica de Catalunya, 08034 Barcelona, Spain; 2 i2Cat Foundation, 08034 Barcelona, Spain

**Keywords:** genetic algorithm, IEEE 802.11ah, RAW, Wi-Fi HaLow

## Abstract

The IEEE 802.11ah standard is intended to adapt the specifications of IEEE 802.11 to the Internet of Things (IoT) scenario. One of the main features of IEEE 802.11ah consists of the Restricted Access Window (RAW) mechanism, designed for scheduling transmissions of groups of stations within certain periods of time or windows. With an appropriate configuration, the RAW feature reduces contention and improves energy efficiency. However, the standard specification does not provide mechanisms for the optimal setting of RAW parameters. In this way, this paper presents a grouping strategy based on a genetic algorithm (GA) for IEEE 802.11ah networks operating under the RAW mechanism and considering heterogeneous stations, that is, stations using different modulation and coding schemes (MCS). We define a fitness function from the combination of the predicted system throughput and fairness, and provide the tuning of the GA parameters to obtain the best result in a short time. The paper also includes a comparison of different alternatives with regard to the stages of the GA, i.e., parent selection, crossover, and mutation methods. As a proof of concept, the proposed GA-based RAW grouping is tested on a more constrained device, a Raspberry Pi 3B^+^, where the grouping method converges in around 5 s. The evaluation concludes with a comparison of the GA-based grouping strategy with other grouping approaches, thus showing that the proposed mechanism provides a good trade-off between throughput and fairness performance.

## 1. Introduction

Wi-Fi HaLow is the certification for products supporting the IEEE 802.11ah standard [1]. This technology represents the response of the IEEE 802.11 Working Group to the increasing connectivity demands of the Internet of Things (IoT). Although the standard was published in 2017, it was not until November 2021 that the Wi-Fi Alliance introduced the Wi-Fi HaLow certification program. Those delays along with the fact that other competing technologies are already well-established on the IoT arena (e.g., LoRaWAN, NB-IoT, Sigfox, etc.), have hampered a wide adoption of the IEEE 802.11ah. Nevertheless, the first certified devices are starting to reach the market, and some studies forecast an increasing interest in this technology [2]. 

Similarly to other IEEE 802.11 technologies, IEEE 802.11ah uses an orthogonal frequency-division multiplexing (OFDM)-based physical layer (PHY), and a carrier-sense multiple access with collision avoidance (CSMA/CA) medium access control (MAC). However, IEEE 802.11ah introduces new features that allow this technology to reach longer ranges than a typical wireless local area network (WLAN), to support thousands of connected devices per access point (AP), and to improve energy efficiency [3].

A longer range is achieved by the use of a lower frequency band below 1 GHz, which introduces lower propagation losses than the 2.4 and 5 GHz bands used in WLANs. Moreover, the support of narrower transmissions (from 1 to 16 MHz, in contrast to the range between 20 and 160 MHz of mainstream IEEE 802.11ax), along with a new and more reliable modulation and coding scheme (MCS) extend the range of a Wi-Fi HaLow network beyond 1500 m while offering a capacity above 100 kbps [3]. Note that other IoT technologies, such as LoRaWAN or Sigfox, cover several kilometers at the cost of a limited capacity (in the scale of 1 kbps), while IEEE 802.11ah’s PHY supports data rates from 150 kbps to 346 Mbps (using multiple antennas-MIMO).

Another focus of the IoT communication is on low power consumption. Further energy savings are, indeed, the target of several new features of the IEEE 802.11ah. For example, the new standard allows longer sleeping periods, helping devices to save more energy while inactive (from several hours of sleep in legacy Wi-Fi, to the year scale in IEEE 802.11ah). IEEE 802.11ah also introduces more efficient frame exchanges and a reduced overhead, which make transmissions more energy-efficient. 

IoT-enabling technologies are also expected to provide connectivity to a large number of devices. Legacy IEEE 802.11 supports up to 2007 associated stations (STA) per AP. Having a longer range and, therefore, covering a larger area, an IEEE 802.11ah AP serves a larger number of STAs. IEEE 802.11ah redefines the Association Identifier (AID), a unique number assigned to each associated STA, to allow up to 8191 STAs per AP. However, note that increasing the number of STAs in a CSMA-based network implies an increased collision probability, which can dramatically degrade the performance with only a few tens of STAs [4]. For example, 250 IoT devices, all transmitting data to a Wi-Fi HaLow AP (all devices use 2 MHz, MCS 5, and one spatial stream) could obtain an aggregate throughput of around 1.5 Mbps; with 500 connected devices, throughput drops to 1.1 Mbps; with 1000 devices, only 0.6 Mbps are achieved, due to the increased number of collisions and resulting retransmissions. In order to reduce the harmful effects of an excessive contention, the IEEE 802.11ah introduces the Restricted Access Window (RAW) mechanism.

With RAW, the AP coordinates the uplink channel access of STAs by defining time intervals in which specific groups of devices are given exclusive access of the shared medium. In this way, the channel access becomes a hybrid between TDMA and CSMA. The benefits of RAW are twofold: (i) collision probability is reduced since only a limited number of STAs contend for the channel during their assigned time window; and (ii) STAs can safely remain in power saving states for longer while they wait for the assigned time slot. On the other hand, RAW poses an important challenge that is left open in the IEEE 802.11ah specification: efficient grouping of STAs. This challenge deals with choosing a number of RAW groups, and selecting the STAs that will be grouped together. Following the numerical example of the previous paragraph, just by defining four equally-sized groups in the case of 1000 active devices, throughput is increased from 0.6 to 1.5 Mbps. In practice, however, devices are not homogeneous and, therefore, more sophisticated grouping strategies are required. For a small amount of STAs, the optimal grouping can be obtained following a simple exhaustive search, in which all the possible grouping configurations are evaluated in a reasonable amount of time. The grouping that maximizes a pre-defined objective function (i.e., fitness function for a genetic algorithm) is then chosen as the optimal RAW setting. However, note that with hundreds or even thousands of STAs, the number of possible groupings is unwieldy (intractable, in practice) and, therefore, an intelligent algorithm, capable of providing (near) optimal grouping decisions within a reasonable time is needed to make the most of the RAW mechanism.

In this paper, we propose a genetic algorithm (GA) specially adapted for tackling the STA grouping problem in IEEE 802.11ah networks with heterogeneous STAs. GAs [5] show, in general, a wide applicability and, in this particular case, provide an intuitive approach to the problem (because of the natural relationship built between groups of STAs and the conceptually simple mechanism of an evolving population), are capable of dealing with very large search spaces (e.g., assuming *G* STAs and a maximum of *R* groups, the number of possible groupings is *R^G^/R!*), and are easily parallelizable, which could be used to take advantage of the multi-cored CPUs present in many modern embedded devices (e.g., Wi-Fi APs). Moreover, GAs are robust to noise and uncertainty because they search for solutions in a probabilistic manner. This means that they can find good solutions even if the problem is noisy or even uncertain; note that the Wi-Fi environment is inherently noisy due to the effects of propagation, interference and mobility of devices.

After detailing the RAW mechanism and discussing the related works in Section 2, our proposed algorithm is described in Section 3, where the implementation details of all the stages of a GA are discussed. As a second contribution, in Section 4 the GA’s hyper parameter tuning is provided; that is, all the design choices are tested to obtain the best performance of the GA. Once the algorithm is optimized at the end of Section 4, the performance of our GA’s decisions is compared with other grouping strategies. Finally, conclusions are provided and possible future works are discussed in Section 5.

The contributions of this paper are as follows:

1. To propose a genetic algorithm adapted for managing the grouping in IEEE 802.11ah networks operating under the RAW mechanism;
2. To provide the tuning and validation of GA parameters (including all the GA phases) to obtain the best performance of the algorithm;
3. To propose a fitness function that reduces the computational time of the algorithm;
4. To evaluate the GA-based grouping proposal using a more constrained device, a Raspberry Pi 3B+;
5. To provide a comparison of the proposed GA-based grouping strategy with other grouping methods.

## 2. Related Work on RAW Station Grouping

This section first reviews the RAW mechanism and different seminal works that provide a general perspective on its operation and performance. Then, the study focuses on the literature related to the specific challenge of RAW grouping and, more precisely, on those approaches seeking throughput and fairness improvements. We discuss their limitations and how our solution addresses these shortcomings. We also highlight the aspects of our proposed solution that are adopted from previous works.

The RAW mechanism is one of the most relevant new features included in the IEEE 802.11ah specification, and it is known to improve throughput, latency and energy efficiency in dense networks [6]. With RAW, an IEEE 802.11ah AP limits the number of stations that contend for the channel during a given time slot by splitting the airtime into different intervals. Some of those intervals are assigned exclusively to a specific group of STAs (RAW groups), while others can be used without restrictions, following IEEE 802.11’s traditional CSMA/CA. Although, in a way, it preserves the original contention-based random access, the RAW signifies a paradigm shift in the IEEE 802.11’s MAC, which moves towards a more centralized access where the AP distributes airtime resources.

**Figure 1:**
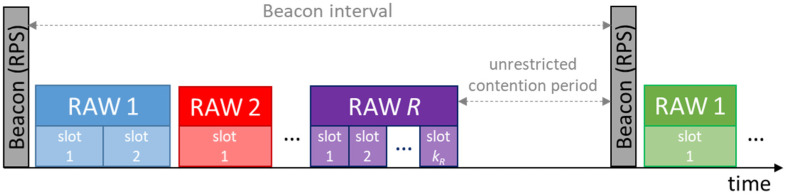
Example of a distribution of the airtime between consecutive Beacons using IEEE 802.11ah’s RAW.

The AP decides how the airtime is shared among the associated STAs and announces its distribution in the RAW parameter set (RPS) information element of Beacon frames. The RPS specifies which STAs belong to which group (using STAs’ AID), the start time and the duration of each RAW. Note that a RAW is further divided into one or more fixed-length slots, and that STAs assigned to a given RAW group are evenly distributed over those slots. The RPS also includes the number of slots and the slot duration of each RAW. Consequently, STAs use contention outside RAWs, and also to access through their assigned RAW slot, which is shared with a reduced number of STAs (if any). Those two types of access follow independent backoff rules (i.e., a backoff function used inside the assigned RAW slot, and another backoff function used outside the slot). During other groups’ RAWs, STAs can remain in a power saving state. Figure 1 depicts a possible distribution of the airtime between two consecutive Beacon frames containing *R* RAW groups, and wherein each RAW *r* is divided into *k_r_* equally-sized slots.

The tuning of the RAW configuration, and more precisely, the STA grouping problem, has received the attention of several research works. In [7], authors thoroughly survey the published works on STA grouping, categorizing the different proposals based on their optimization goals. Although the algorithm proposed in this paper would fall within the categories of throughput-oriented and fairness-oriented algorithms, it is worth noting that a GA can be easily adapted to optimize any other metric, provided that the fitness function (cf. Section 3.1) reliably reflects the impact of the decided RAW configuration on the targeted metric.

With the goal of improving channel utilization (and thus, capacity), the work in [8] proposes a STA grouping scheme seeking load balancing among RAW groups. This approach is based on integer programming and works under the assumption that the number of RAW groups is fixed and that the AP knows the STAs’ offered traffic behavior. In [9], the same authors refine their proposal and further derive a regression-based model to estimate the contention success probability. Knowing STAs’ traffic in advance is not trivial, but there are different mechanisms that would allow the AP to obtain a good estimation. For example, STAs could describe their traffic needs using IEEE 802.11’s Traffic Specification (TSPEC) element. In [10], authors propose the Traffic-Aware RAW Optimization Algorithm (TAROA), which tries to predict the inter-packet time of STAs by analyzing that STA’s past transmissions. In [11], they proposed a more accurate traffic estimation method by exploiting the “More Data” flag of the IEEE 802.11’s header for the Enhanced-TAROA (E-TAROA). The authors in [12] also predict packet transmissions, in this case by averaging the observed inter-packet times of active STAs and assuming a periodic behavior. They also define a contention phase (for any STA’s first transmission) and a reservation phase, where STAs expected to have pending frames transmit without contention. These works support our assumption that the AP could know the set of STAs with pending traffic for the following Beacon interval. 

In [13], authors argue that in order to improve throughput, the duration of RAW slots should be set as a function of the number of STAs in the group. Although their idea to set different durations for the time slots in a RAW is not compatible with the IEEE 802.11ah specification (slots within a RAW must have the same duration), a similar approach is followed by configuring one-slot RAWs, the duration of which is proportional to the number of assigned STAs.

Note that most of the prior work is limited to homogeneous STAs, that is, all STAs use the same MCS, same transmitted power, and even the same packet size. In practice, STAs are located at different distances from the AP and, hence, they will use different MCS (i.e., different PHY rate). The coexistence of STAs using different MCS impacts fairness and the available throughput [14] and should therefore be considered when configuring the RAW. In [15] and [16], authors deal with those heterogeneous scenarios by grouping STAs with the same PHY rate. However, as discussed in Section 4.1, mixing STAs with different PHY rates in a particular way can result in a better fairness and throughput. With a focus on fairness, authors in [17] group STAs according to their traffic profile (inter-packet interval and packet size) and propose a dynamic configuration of STAs’ contention window, which would require changes to the standard backoff mechanism. Another work seeking to maximize throughput and fairness, but also considering hidden nodes is presented in [18], wherein the authors propose an Ant Colony algorithm to solve a Max-Min fairness optimization. As with [8], the main drawback of this approach is that the number of groups *R* is fixed; we argue that *R* is one of the key parameters of the RAW configuration (cf. Section 4.1).

All in all, compared to other capacity and/or fairness driven approaches found in the literature, we argue that the GA-based solution proposed in this paper has several key advantages. It avoids the limiting assumption of a fixed number of groups, and supports the presence of heterogeneous (in terms of PHY rate and packet size) STAs, while staying fully IEEE 802.11ah compliant. On the other hand, our solution assumes that the scheduler knows which STAs will require uplink resources in the next interval, an assumption that has been supported by previous research in this field.

## 3. Problem Definition and Methodology: A Grouping Strategy for IEEE 802.11ah Based on a Genetic Algorithm

In this section, our proposal of a grouping strategy based on a genetic algorithm is presented. We introduce the basics of the genetic algorithm used in this research work, describe the different phases involved in the algorithm, and discuss the alternatives considered in this evaluation.

Based on Charles Darwin’s theory of natural evolution, genetic algorithms are heuristic search algorithms that try to reflect the process of the natural evolution, where the fittest individuals are selected out of the population for reproduction, and produce the offspring of the next generation. 

The process starts with the selection of the fittest individuals from an initial population. These individuals will produce offspring, and the characteristics of the parents will be inherited by the next generation. This essence can be applied in a station grouping strategy, where a set of initial individuals (a set of groups of stations) will be genetically evolved in order to select the best set according to given criteria. A genetic algorithm includes five phases, i.e., initial population, fitness function, selection of parents, crossover function, and mutation phase, which are detailed in the following.

**Figure 2:**
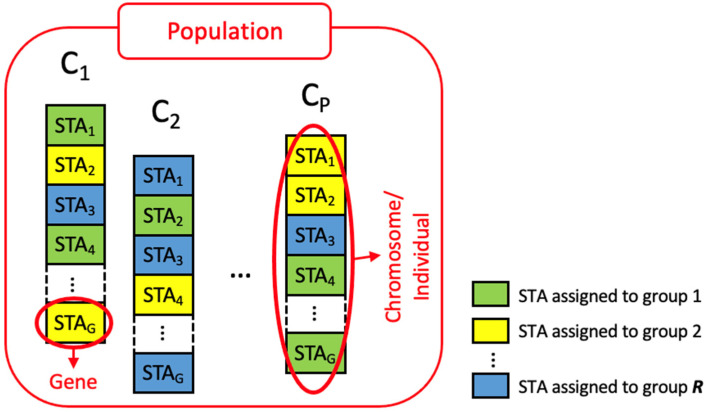
Modelling IEEE 802.11ah STAs grouping as a genetic optimization problem.

The GA must start with an initial set of *P* individuals called *initial population*. Each *individual* represents a valid solution to the problem to be solved, and is characterized by a set of *G* parameters, known as *genes*. In similitude with Darwin’s theory, an individual is also named *chromosome*. In our case, each of the *P* chromosomes (*C_i_*) of a population consists of a set of *G* IEEE 802.11ah STAs, and each STA is assigned to one of *R* possible groups. The group assigned to each STA represents a gene, as depicted in Figure 2.

Next, the fitness function (Phase 2) is applied to each chromosome *C_i_* of the population, which is in charge of evaluating how fit an individual is, i.e., its ability to compete with other individuals. The output of this method is a fitness score for each individual, and determines the chances of an individual to be selected for reproduction. The larger the value, the fitter the individual and, thus, the higher the probability of the individual to be chosen for offspring production. The fitness function defined for our grouping strategy is presented in Section 3.1 as a function of the expected system throughput and fairness.

Another function will choose the fittest individuals and use their genes to produce a new population (Phase 3). Two or more pairs of individuals (known as parents) are selected based on their fitness scores to breed the new individuals that will populate the next generation. There are different methods available for parent selection. Section 3.2 discusses the different methods considered in the evaluation.

The crossover function (Phase 4) defines the method for parent reproduction, i.e., the form in which the genes of each parent are selected for producing the next generation of individuals. Again, there are different crossover functions. The methods used in the present evaluation are discussed in Section 3.3.

Finally, in the mutation phase (Phase 5), some of the genes of the newly created offspring may be altered. Mutation is needed to keep a healthy diversity in the population, and to prevent a premature convergence of the algorithm due to a local optimum. As in the case of parent selection and crossover function, there are different alternative mechanisms for applying mutation; they are discussed in Section 3.4.

When run, the GA loops between Phases 2 and 5. The algorithm terminates when the population has converged, i.e., the generated offspring is not significantly different from the previous generation. Alternatively, a stop condition may be set to stop the algorithm at a specific time or event. Algorithm 1 summarizes the GA working procedure.
**Algorithm 1.** GA working procedure.  **Initialize** population; **Apply fitness** function for population evaluation; Generation = 0; **while termination** criterion is not satisfied { Select good individuals through **parent selection** function; Parent reproduction through **crossover** function; Apply **mutation** function; **Apply fitness** function for population evaluation; Generation = Generation + 1; } **return** the best individual shown during the evolution;

### 3.1. Fitness Value Computation

Targeting an efficient use of the spectrum, the fitness value used in the proposed GA considers the system’s potential throughput. As a starting point, the throughput computation given by the Bianchi model for multi-rate and heterogeneous environments [19] adapted to IEEE 802.11ah PHY and MAC [20] is used. This model concludes with the following expression for total system throughput, *S*:

(1) $$S=\overset{R}{\underset{r=1}{\sum }}\frac{d_{r}*\sum_{k=1}^{k_{r}}\frac{S_{slot\_k}}{k_{r}}}{d_{total}}\;$$

where *R* is the number of RAW groups, each of them having *k_r_* slots, *d_r_* corresponds to the duration of the *r_th_* RAW group, and *d_total_* represents the total duration of all *R* RAW groups together. *S_slot_k_* stands for the throughput corresponding to the *k_th_* slot; its computation is taken from the Bianchi model for multi-rate environments [19]. The reader is referred to [20] for further detail on the computation of *S*.

However, a fitness function focused solely on maximizing throughput would end up marginalizing slow STAs, which will have less opportunities to transmit their data (e.g., STAs receiving a poor signal from the AP and, hence, using a more reliable, but slower MCS). To prevent the possible starvation of slow STAs, a measure of fairness is also considered within the proposed fitness function. The fairness metric determines whether STAs are receiving a fair share of system resources. In our fitness function, the well-known Jain Fairness index [21] is used for fairness calculation. 

In this way, the fitness value (*V*) is obtained as the combination of the total system throughput (*S*) and the fairness (*F*), i.e., *V = S ∗ F*. 

### 3.2. Parent Selection

There are different functions in the literature for the selection of parents. In the following, the most relevant ones are presented, which have been considered for our evaluation. The *Fitness Proportionate Selection* (FPS) method, also known as *roulette wheel selection* [22], consists in choosing two parents among all the individuals based on a probability, proportional to their fitness value. Each possible individual is assigned a slice of the wheel of a size based on its corresponding fitness value. Then, a random selection is done for choosing each of the two parents (i.e., the roulette wheel spins twice). This method includes a variation called *Stochastic Universal Sampling* (SUS) [23], which chooses the two parents with a single spin of the wheel; one parent is randomly chosen following the aforementioned FPS approach, and the other is the diametrically opposite element of the wheel. The advantage of SUS is the lower computational cost with respect to FPS. On the other hand, there is the *Rank Based Selection* (RBS) method [24], which consists in performing a ranking with all the individuals based on their corresponding fitness value. Then, a probability value is given to each individual considering its location in the ranking, i.e., the higher the ranking position, the larger the probability value assigned. Afterwards, a random selection is performed for choosing each of the parents. Typically, two parents are considered for producing the next generation of individuals, following any of the methods mentioned above. However, more than two parents can be selected. As discussed in reference [25], using more than two parents does not clearly improve the results, while it leads to larger computational costs. Finally, we consider a method by which all *P* individuals participate in the breeding, thus providing each individual a number of genes to the new offspring, proportional to its fitness value. 

### 3.3. Crossover Function

With regards to crossover functions, several alternatives can be found in the literature, describing different ways of mixing the parents’ genes to create new offspring. In the following, the methods considered in our evaluation are described. The *one-point crossover* or *single-point crossover* [26] consists in choosing randomly a crossover point on both parents. This can be implemented by randomly selecting a gene number as the crossover point; the genes to the left of the crossover point (i.e., smaller gene number) on one parent are combined with the genes to the right of the point on the other parent. The *multi-point crossover* [26] extends the previous approach by using two crossover points randomly chosen over both parents. The genes in between the two crossover points are swapped between the parents in order to create the offspring. This method can be extended to more than two crossover points. With the *ring crossover* [27], the genes of both parents are concatenated one after the other, and then organized in the form of a ring or circular buffer (i.e., the last gene of the second parent is followed by the first gene of the first parent). Next, the ring is cut in half at a random point (cutting point). Each half shows a different combination of its parents’ genes and constitutes a new offspring. In the *uniform crossover* [26], each gene is chosen from either parent with equal probability, i.e., 50%. Moreover, it is also common to preserve the best individuals of one generation and pass them on to the next. The number of individuals that preserve their genes after a new generation is born is managed by another configurable parameter called *pressure*.

### 3.4. Mutation Function

The mutation step is applied over the new generation of individuals that are not under the pressure parameter. As with previous phases, there are several options in the literature for implementing the mutation phase. In this section, the most relevant ones are exposed, which have been considered in this evaluation. The *Partial Shuffle Mutation* (PSM) [28] mutates or preserves each gene of an individual based on a predefined mutation probability. A variation of this method limits the mutation to only one randomly selected gene. Following the *Reverse Sequence Mutation* (RSM) [28], two points are randomly chosen on the individual. The segment of genes between the two points is swapped based on a predefined mutation probability. Finally, the *Swap Mutation* (SM) [29] consists in swapping two genes of the individual, chosen at random. Again, the swapping action is performed under a predefined mutation probability.

## 4. Evaluation

For our evaluation, we have developed the proposed GA-based grouping strategy algorithm for IEEE 802.11ah using Python. In this section, we present the tuning of the GA parameters to get the best fitness result in a reduced amount of time. We use a PC with Intel Core i9 at 3.30 GHz and 62 GB of RAM. After completing the validation of the algorithm, we provide results using a more constrained device, a Raspberry Pi 3B+, that can act as AP, and run the grouping mechanism proposed in this paper.

### 4.1. Initial GA Parameter Setting

First, the initial tuning and validation of the GA is performed, considering a sample scenario with 33 STAs and wherein all 11 MCS are in use (MCS 0 to MCS 10 for the 1 MHz PHY IEEE 802.11ah), that is, three STAs per MCS. The maximum possible number of RAW groups *R* is set to eight. All the individuals are considered as parents, and each individual provides a number of genes to the new offspring proportional to its fitness value. In Section 4.3.1, a detailed evaluation on parent selection methods is shown. Moreover, in this initial parameter setting, a mutation method is considered, in which mutation is performed only on one randomly selected gene of an individual (i.e., PSM mutation applied to one gene), with a mutation probability of 0.2. Later, in Section 4.3.3, the study on mutation methods is presented in depth. The GA is stopped if all the individuals of the population have the same genes, or after 200 loops, if the first condition is not met.

**Figure 3:**
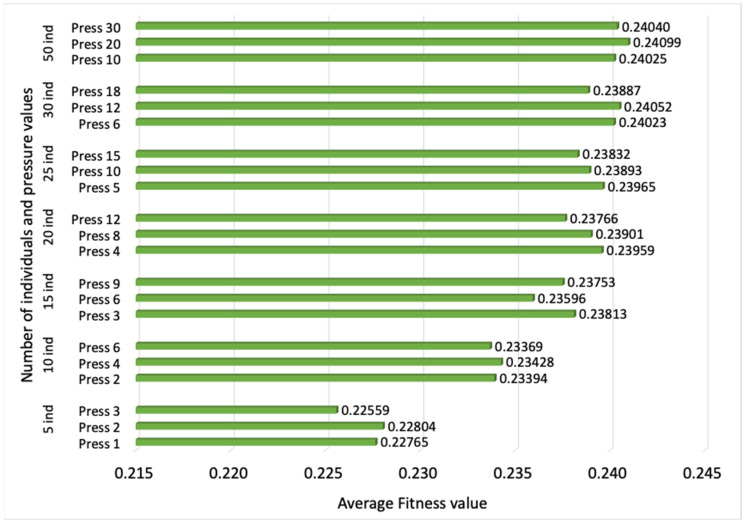
Average fitness value of the best individual for populations built of different number *P* of individuals and pressure values.

**Figure 4:**
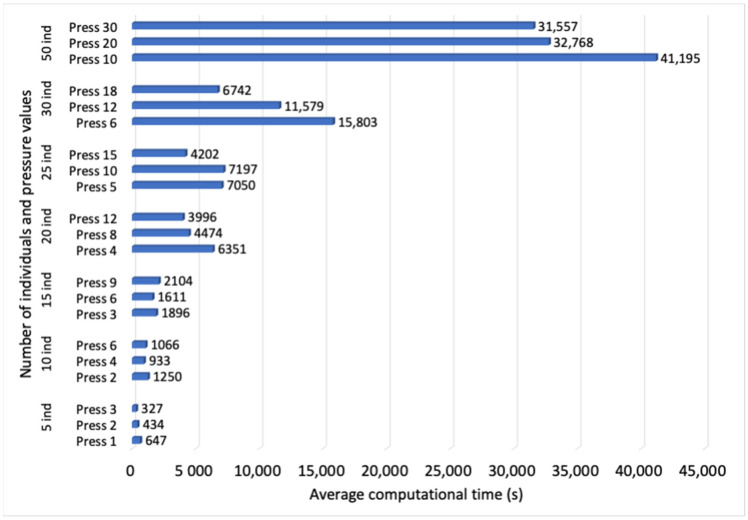
Average computational time (s) for populations built of different number *P* of individuals and pressure values.

The first parameter to evaluate is *P*, the number of individuals that form the population. Populations built of 5, 10, 15, 20, 25, 30 and 50 random individuals are considered. The average fitness value of the best individual in the final population over 10 simulations, and the average computational time, can be observed in Figure 3 and Figure 4, respectively, vs. different pressure values. The results show that an increase in the number of individuals leads to a higher fitness value, but from 20 individuals on, the improvement is slowed down, while the computational time experiences an important rise. In this way, the configuration with *P* = 20 individuals is identified as the most convenient, since computational time is not severely compromised, and the fitness value does not show an important reduction in comparison with the results observed for a larger *P*.

**Figure 5:**
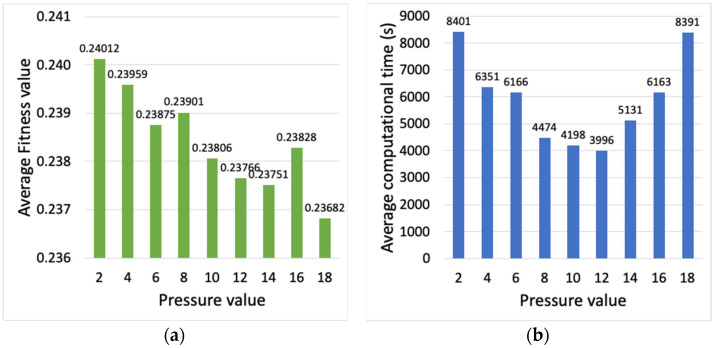
(**a**) Average fitness value of the best individual, (**b**) average computational time (s), vs. pressure.

With the number of individuals set to 20, the next parameter considered is the pressure, with values comprised between 2 and 18. Note that the pressure parameter determines the number of individuals that survive to the next generation preserving their genes intact. Figure 5a,b presents the average fitness value of the best individual and the average computational time, respectively. Note that both the largest and smallest pressure values show high computational time. With large pressure values, a high portion of the new population is kept from the previous one, and thus the algorithm needs more iterations to evolve the population, and concludes with a worse fitness value. On the other hand, a small pressure is helpful to achieve a better fitness but at the cost of longer computational times. A pressure value of 8 is chosen, since it shows a good balance between the two metrics.

**Figure 6:**
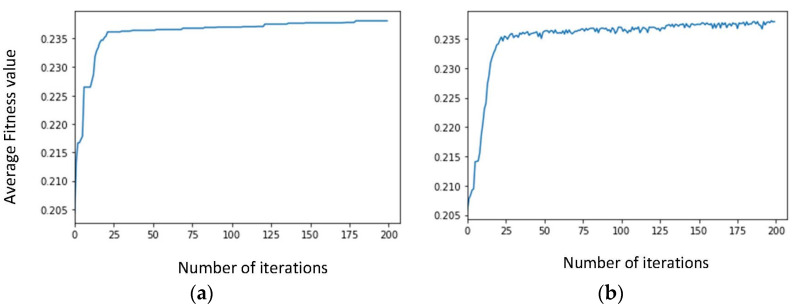
Evolution of average fitness of (**a**) the best individual, (**b**) all the individuals, over the genetic loop, vs. number of iterations-initial stop condition.

**Figure 7:**
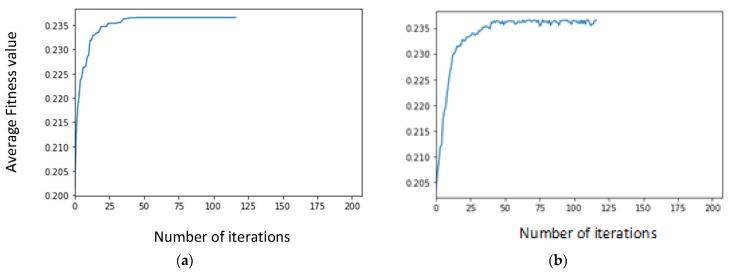
Evolution of average fitness of (**a**) the best individual, (**b**) all the individuals, over the genetic loop, vs. number of iterations-final stop condition.

Next, the adjustment of the stop condition is carried out, with the objective of reducing the computational time of the GA. Unless explicitly mentioned otherwise, in the following, the aforementioned configuration parameters are always considered. Figure 6 shows the evolution of the fitness value when the average over the best individual is considered (Figure 6a), and when the average over all the individuals is taken into account (Figure 6b). After around 80 iterations, the curve starts to flatten, and the improvement is very residual. In consequence, a new stop condition is set up with the GA running for, at least, 80 iterations, after which, the level of improvement between one generation and the next is compared: if for ten non-consecutive iterations the fitness value does not improve 0.05% or more, the GA is terminated. Figure 7 shows the performance for the new stop condition, where it can be observed that GA stops after 120 iterations, with the corresponding reduction in the computational time.

**Figure 8:**
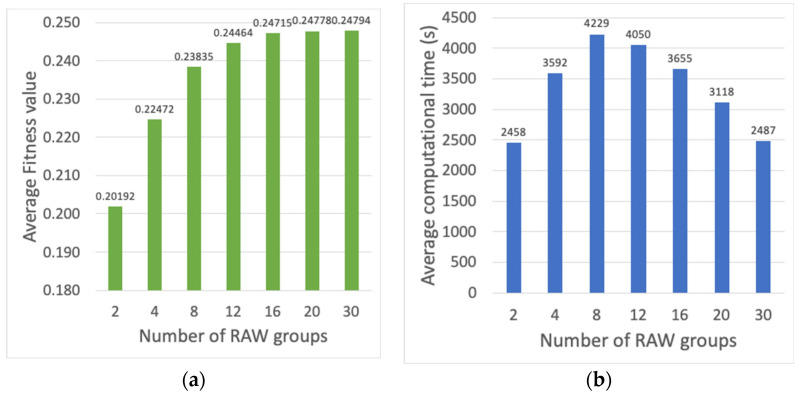
(**a**) Average fitness value of the best individual, (**b**) average computational time (s), vs. number of RAW groups comprised between 2 and 30.

Following, the evaluation is focused on the number of RAW groups. With this objective, scenarios with 2, 4, 8, 12, 16, 20 and 30 RAW groups are considered. From Figure 8a,b, it is observed that using a large number of RAW groups, between 20 and 30, better fitness values with reduced computational time are obtained. However, a large number of groups increased the Beacon overhead. Moreover, following the conclusions from reference [6], it also leads to stations experiencing larger latencies between transmissions. Because of the aforementioned reasons, a large number of RAW groups is avoided, and the use of 12 groups is chosen as the most suitable option, i.e., it offers a good tradeoff between fitness value and Beacon overhead and latency.

**Figure 9:**
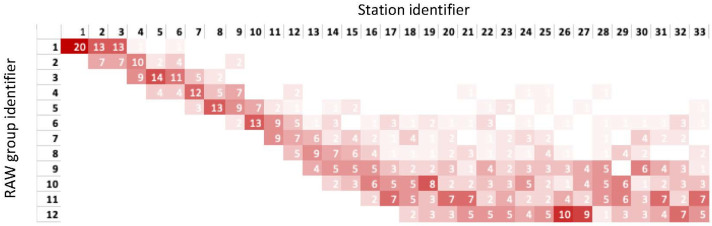
Heat map representing the frequency with which the GA assigns STAs to different groups (*G* = 33 STAs, *R* = 12 RAW groups).

**Figure 10:**
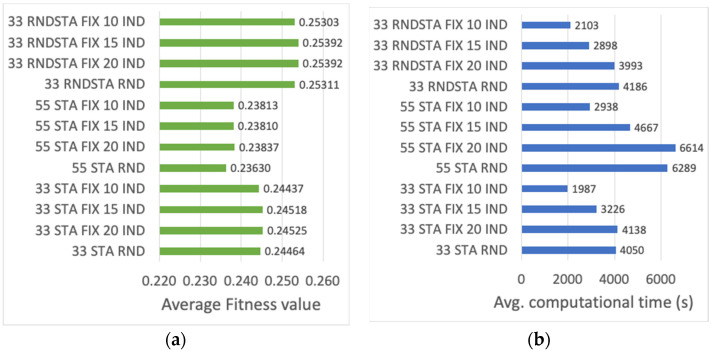
(**a**) Average fitness value of the best individual, (**b**) average computational time (s), for different initial populations.

Finally, the initial population is studied with the objective of finding an initial configuration that maximizes the final fitness value. So far, all the individuals building the initial population have been generated randomly. Thus, the usage of a fixed initial individual is evaluated. In order to identify this individual, the result of 20 independent runs of the genetic algorithm using an initial random population is represented through the heat map of Figure 9. The horizontal axis represents the 33 stations of the scenario, ranked in increased PHY rate order (i.e., STAs 1 to 3 use the slowest MCS, and STAs 31 to 33 the fastest). The vertical axis shows the RAW group identifier, where *R* = 12. The heat map represents the frequency (out of 20 runs) with which each station has been assigned to a given RAW group. It can be observed, that the algorithm groups the stations differently depending on their rate. It is especially notorious how the GA tends to isolate the slowest stations, which are concentrated in the first three groups. Stations with a faster rate are spread over different RAW groups. In other words, according to the GA, the most suitable grouping to maximize throughput and fairness requires that some groups concentrate a few very slow STAs, while other groups contain a larger number of mixed faster stations. Following these conclusions, the genes composing the fixed initial individual are chosen, that is, genes are initially set according to the group assignments most frequently observed, as represented in Figure 9. In order to evaluate the performance of the GA using a fixed initial individual, populations of *P* = 10, 15 and 20 individuals are considered, in scenarios with *G* = 33 and 55 stations (or genes per individual), and with 3 and 5 operating STA per MCS, respectively, and a scenario where the MCS is set at random. The average fitness value of the best individual, and the average computational time, are presented in Figure 10a,b, respectively, for the cases of Table 1. When an initial fixed individual is used, the average fitness value of the best individual and of all the individuals in the final population improves for populations of 15 and 20 individuals. In the case of *P* = 20, however, the increase in computational time is notorious.

Based on the analysis presented in this section, hereafter, an initial population composed of 15 individuals is considered, one of them fixed according to the frequencies observed in Figure 9. Moreover, unless mentioned otherwise, scenarios contain 33 STAs configured with a random MCS, a maximum of 12 RAW groups, a pressure value of 8, and a PSM mutation applied to one gene with mutation probability of 0.2.

### 4.2. New method for Fitness Computation

Through the evaluation performed in previous Section 4.1, high computational time (some thousands of seconds) is observed for the convergence of the GA. The method employed for throughput calculation, which is required for obtaining the fitness value (cf. Section 3.1), accounts for most of the computational time (around 98% of the execution time is used by the fitness function). In this section, an alternative method for throughput calculation is proposed, with the objective of reducing the complexity of the fitness function and, thus, the complexity and computational time of the algorithm.

As discussed in Section 3.1, the Bianchi model for multi-rate environments [19] adapted to IEEE 802.11ah PHY and MAC [20] is employed for throughput estimation. The adapted Bianchi’s model provides an equation system to obtain a STA’s transmission probability (from which the collision probability *P_c_* is obtained as a function of the number of stations *N* per RAW slot, *P_c_ (N)*), which is solved by means of numerical methods thus entailing high complexity. The new proposal for throughput computation simplifies that process by pre-computing the values of the collision probability, and hard-code them within the algorithm. The average number of transmissions of a frame (*R_tx_* in Equation (2)) is obtained directly applying pre-computed *P_c_ (N)*, being *R_tx_* then employed for computing the average time spent in backoff ${\bar{TBO}}_{Rtx}$ during busy periods within a RAW slot (*T_cycle_* in Equation (3)). ${\bar{TBO}}_{Rtx}$ computation is detailed in [30]. Finally, the corresponding throughput *S_slot_k_* for the slot *k* follows Equation (4), the output of which is used in Equation (1) for total system throughput *S* computation:

(2) $$R_{tx}=\frac{1}{1-P_{c\left(N\right)}}$$

(3) $$T_{cycle}={\bar{TBO}}_{Rtx}+N*\left(DIFS+SIFS\right)+\sum_{j=1}^{N}T_{data\_j}+\sum_{j=1}^{N}T_{ack\_j}$$

(4) $$S_{slot\_k}=\frac{\left(1-P_{c\left(N\right)}\right)\sum_{j=1}^{N}L_{j}*8\;}{T_{cycle}}$$

where *DIFS* and *SIFS* correspond to standard values included in the IEEE 802.11ah specification [1], *T_data_j_* stands for the time involved in the transmission of a data frame sent by station *j*, *T_ack_j_* for the duration of an *ACK* frame transmitted by station *j*, and *L_j_* for the payload size in bytes of data frames generated by station *j*.

The fitness with the new computation of throughput is included in the GA, and the algorithm performance is evaluated in terms of convergence time and fitness, with respect to the results observed employing the original fitness calculation. The same scenarios have been used with the original and the new function, and the final population has been obtained after convergence of the GA. Then, the final population in both cases has been evaluated using the original fitness function. The difference of the fitness (per best individual, and per final population) achieved using the new function, compared to the original function is shown in Table 2 for different scenarios. The reduction observed is between 2% and 3%, in the worst case, which results in an acceptable magnitude. On the other hand, Table 3 presents performance with regard to the computational time, which certifies the reduced complexity of the algorithm using the new fitness function proposed. In this case, a reduction of around 10,000 times is observed for the computational time, when using the new fitness function, which more than compensates for the small decrease in final fitness metrics. Accordingly, hereafter, the new fitness computation method is adopted in the proposed GA-based grouping strategy. 

### 4.3. Tuning of Parent Selection, Crossover and Mutation Methods

In this section, a comparison of different alternatives is provided with regard to parent selection, crossover and mutation methods.

#### 4.3.1. Parent Selection

**Figure 11:**
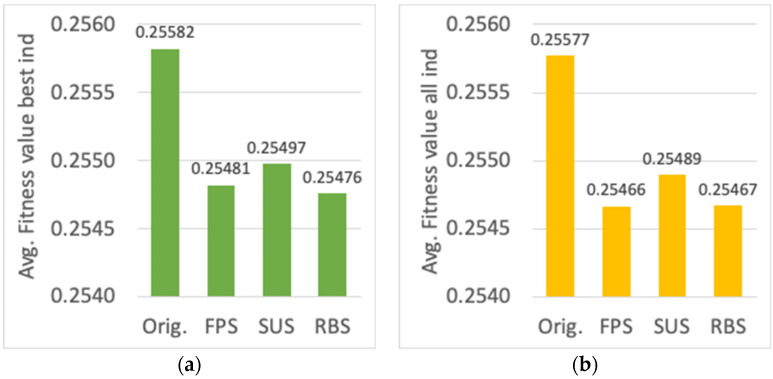
Average fitness value of (**a**) the best individual, (**b**) all the individuals, for different parent selection algorithms.

**Figure 12:**
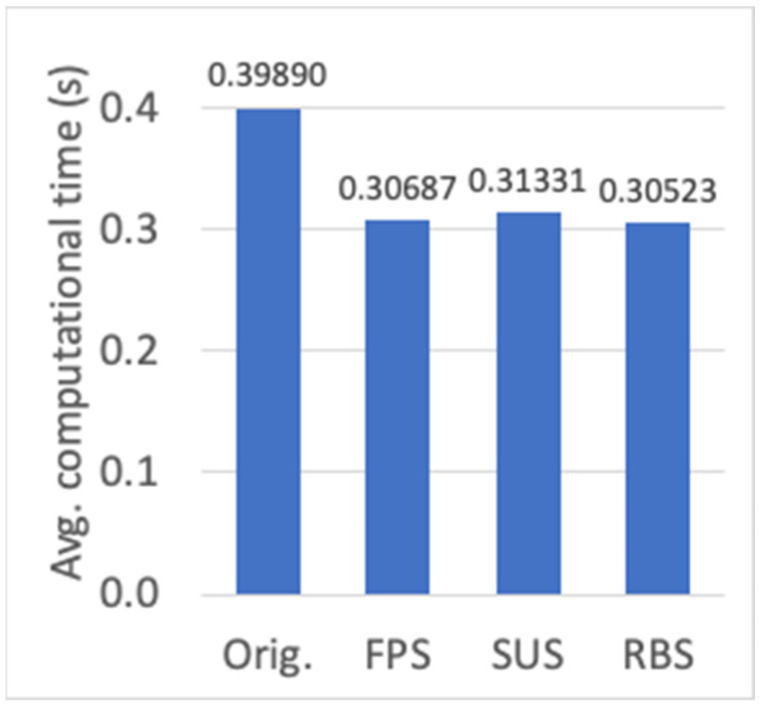
Average computational time (s) for different parent selection algorithms.

First, the evaluation for different parent selection mechanisms is shown (cf. Section 3.2). Results for the average fitness value of the best individual, the average fitness value of all the individuals in the final population, and the average computational time, are presented in Figure 11a,b and Figure 12, respectively. Although a slight decrease in the fitness value can be observed (around 0.4%) for FPS, SUS and RBS methods with respect to the one used originally (all the individuals are considered parents, thus providing each individual a number of genes to the new offspring proportional to its fitness value), there is a significant decrease in the computational time (around 20%). Among FPS, SUS and RBS, the latter shows the lowest computational time, thus it has been chosen as the parent selection method.

#### 4.3.2. Crossover Evaluation

**Figure 13:**
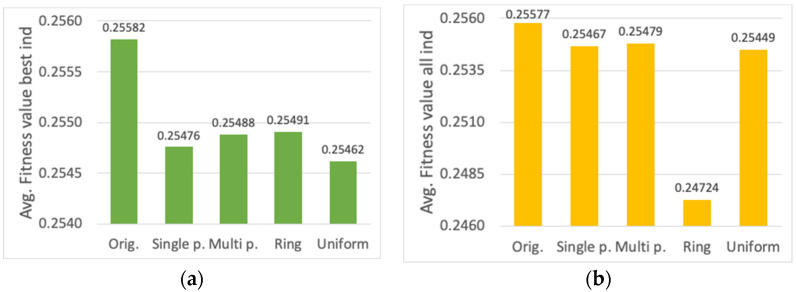
Average fitness value of (**a**) the best individual, (**b**) all the individuals, for different crossover methods.

**Figure 14:**
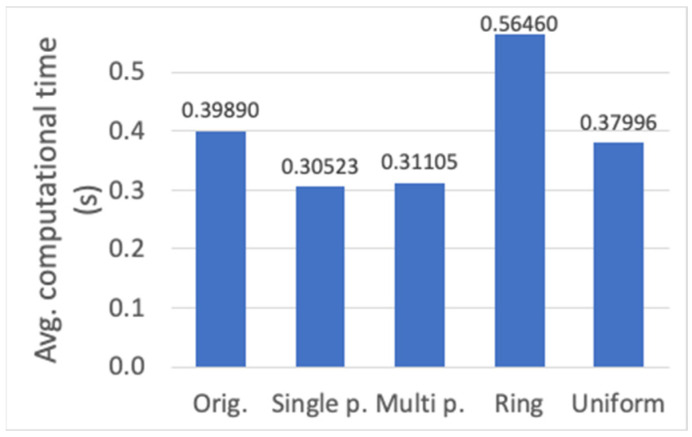
Average computational time (s) for different crossover methods.

Next, the analysis of crossover alternatives is performed (cf. Section 3.3). Again, evaluation results are shown for the average fitness value of the best individual, of all the individuals in the final population, and the average computational time (Figure 13a,b and Figure 14, respectively). The different methods are compared with the original mechanism used, in which all the individuals provide certain number of genes to the new offspring proportional to their corresponding fitness value. The *ring* crossover method shows the worst performance for both fitness and computational time performance. On the other hand, the *single-point*, *multi-point* and *uniform* crossover functions only present a slight decrease in the fitness value (around 0.5%) with respect to the original method, whereas the reduction in the computational time is remarkable (around 23%). Among the three alternatives, the *single-point* method is chosen, which obtains the lowest computational time.

#### 4.3.3. Mutation Evaluation

**Figure 15:**
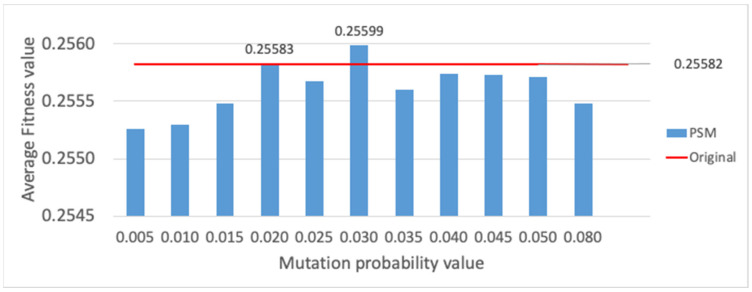
Average fitness value of the best individual for PSM method vs. different mutation probability values.

**Figure 16:**
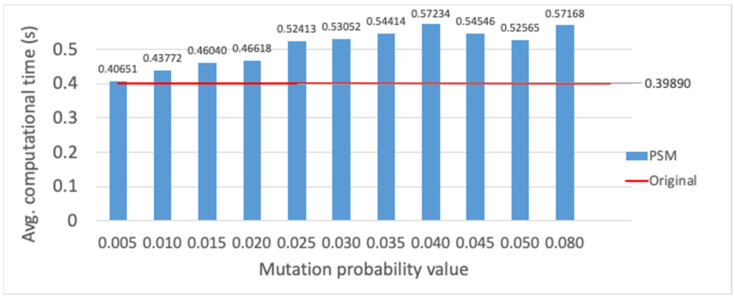
Average computational time (s) for PSM method vs. different mutation probability values.

**Figure 17:**
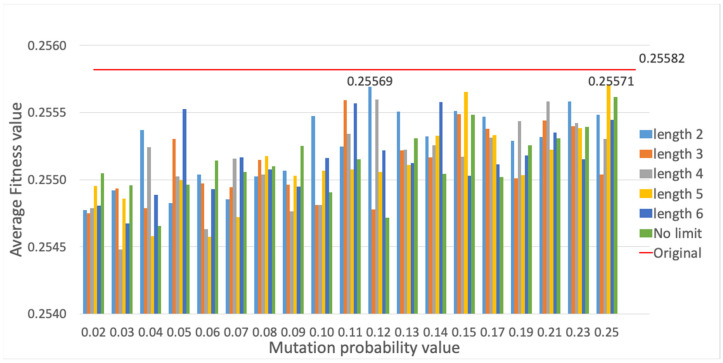
Average fitness value of the best individual for SM method vs. different mutation probability values.

**Figure 18:**
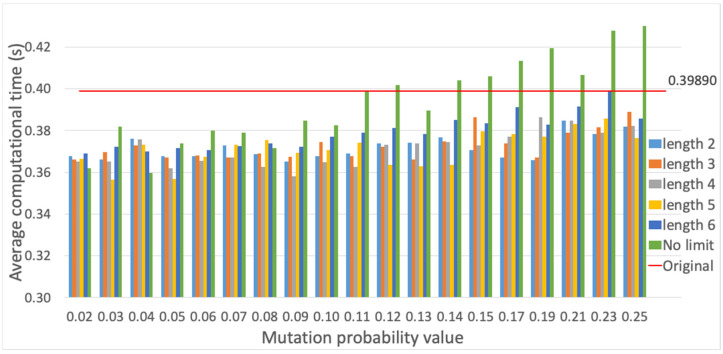
Average computational time (s) for SM method vs. different mutation probability values.

Finally, the evaluation of different mutation functions is provided (cf. Section 3.4), which are compared with the method originally employed, where mutation is performed only on one gene randomly selected per individual. Each mutation alternative is evaluated, i.e., PSM, SM and RSM methods, for different mutation probability values, and evaluation results are presented for the average fitness value of the best individual and the average computational time. The PSM evaluation is shown in Figure 15 and Figure 16. Employing a mutation probability of 0.3, the fitness value improves around 3% over the result obtained with the original method. However, computational time experiences a high increase. Thus, PSM is discarded as mutation alternative. Secondly, results are provided for SM in Figure 17 and Figure 18. Various lengths are considered for the segment of genes; recall that, employing SM, two genes of the individual inside the segment are randomly chosen and swapped. The configuration showing the best performance corresponds to a segment length of 5 and a mutation probability of 0.25. In this case, the fitness value is slightly reduced with respect to the original method, but the computational time obtains a non-negligible reduction of around 6%.

**Figure 19:**
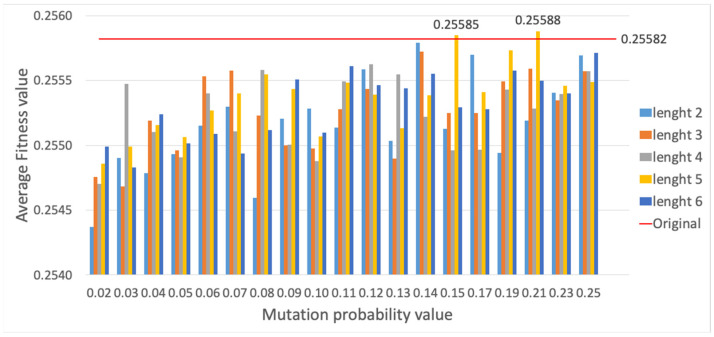
Average fitness value of the best individual for RSM method vs. different mutation probability values.

**Figure 20:**
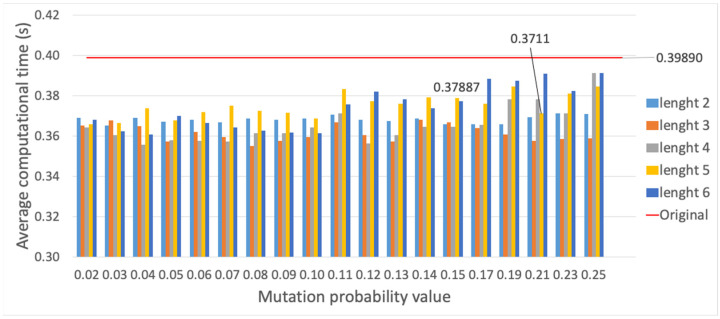
Average computational time (s) for RSM method vs. different mutation probability values.

Lastly, results are presented for RSM in Figure 19 and Figure 20. Again, different lengths for the segment of genes to be swapped have been considered; remind that RSM consists of randomly choosing two points on the individual, and swapping the segment of genes between these two points. It can be observed that the configuration with a segment length of 5 and mutation probability of 0.21 leads to a better fitness and computational time performance, thus improving previous SM results. In this way, the RSM method is selected among the three alternatives.

### 4.4. Test over Raspberry Pi

After completing the validation of the genetic algorithm, results are provided using a more constrained device, a Raspberry Pi 3B^+^ (1.4 GHz processor and 1 GB of RAM), running the grouping mechanism proposed in this paper. Performance results with regard to computational time are summarized in Table 4. The important improvement achieved in complexity by the introduction of the new method for fitness computation discussed in Section 4.2 is confirmed. With the new method, computational time is reduced from around 9.5 h to 7.7 s. Secondly, the selection of the new crossover function (single-point method) provides a non-negligible reduction from 7.5 s to 5.6 s. Finally, with the mutation method selected (RSM), computational time is reduced even further to 5.1 s. This time value allows the usage of the GA and grouping mechanism proposed in a real and dynamic environment, where the algorithm needs to be run in an AP regularly, without disturbing the current functions of the AP.

### 4.5. Grouping Strategy Comparison

**Figure 21:**
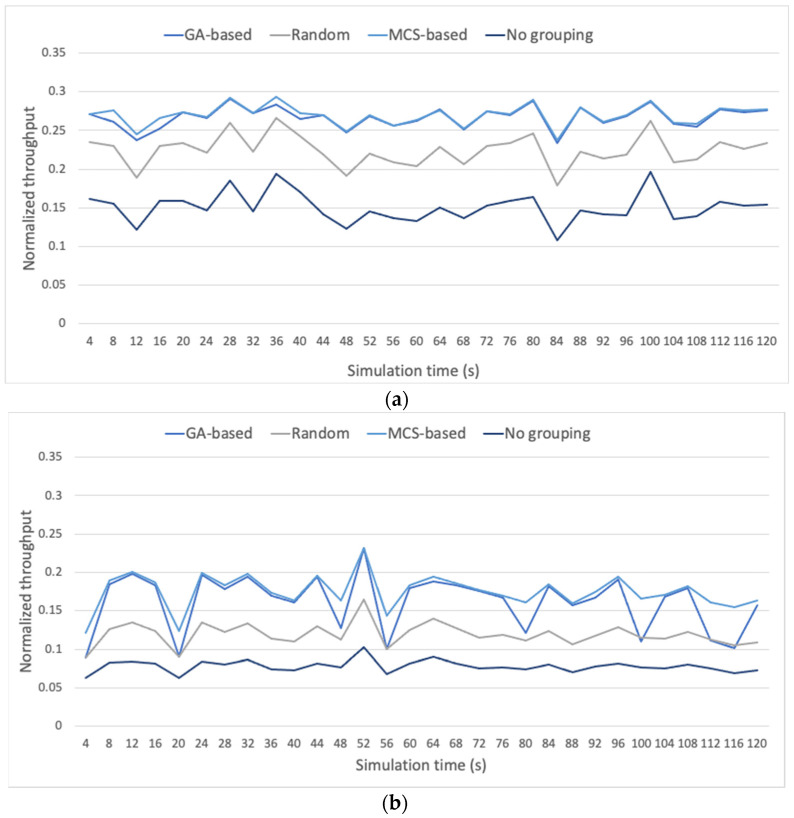

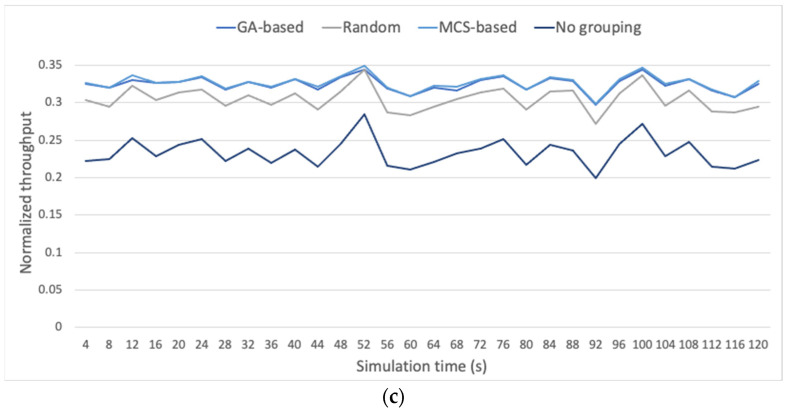
Throughput performance for scenario with STAs with (**a**) MCSs uniformly distributed, (**b**) MCSs distributed with 50% probability of being the slowest MCS (i.e., MCS 10), and 50% probability of being any of the remaining MCSs (MCS 0 to MCS 9), and (**c**) MCSs distributed with 50% probability of being the fastest MCS (i.e., MCS 9), and 50% probability of being any of the remaining MCSs.

**Figure 22:**
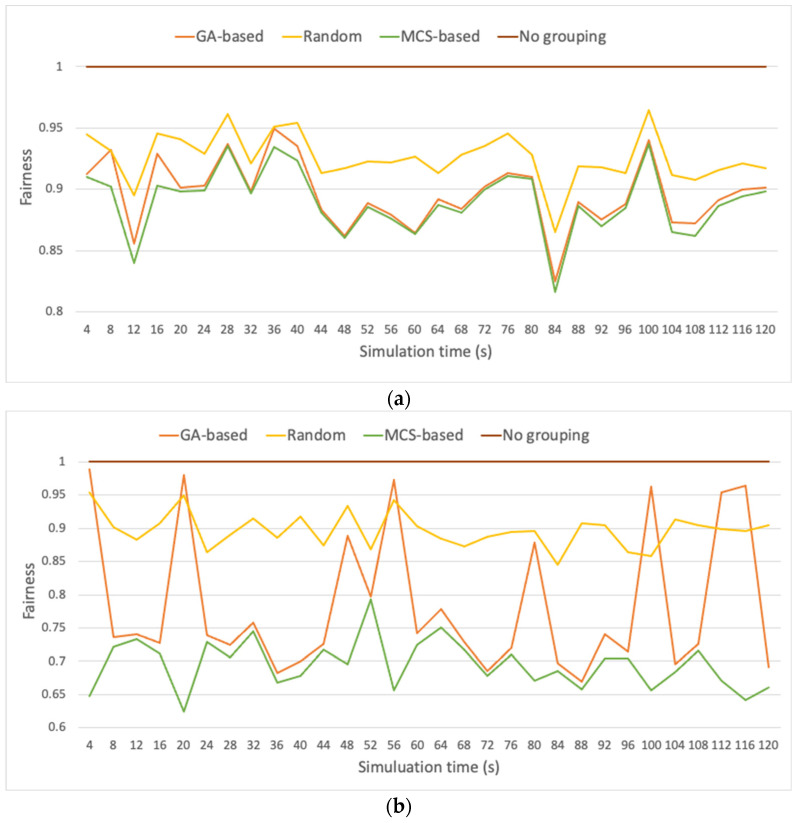

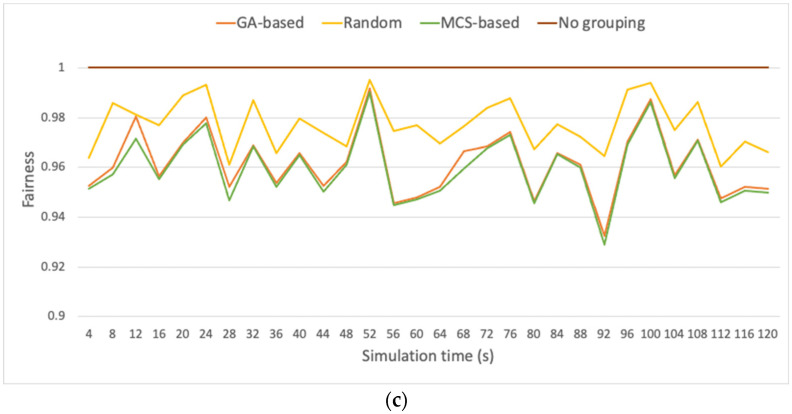
Fairness performance for scenario with STAs with (**a**) MCSs uniformly distributed, (**b**) MCSs distributed with 50% probability of being the slowest MCS (i.e., MCS 10), and 50% probability of being any of the remaining MCSs (MCS 0 to MCS 9), and (**c**) MCSs distributed with 50% probability of being the fastest MCS (i.e., MCS 9), and 50% probability of being any of the remaining MCSs.

To complete the evaluation section, a comparison of the GA-based grouping strategy with other grouping methods is provided. A large scenario composed of 1800 STAs is considered, each one operating under a duty cycle of 2.8% [31]. The Beacon interval is of 4096 ms [32], and a total simulation time of 2 min is considered. The GA-based strategy is compared against three strategies commonly used as benchmark in the related literature [7]: (i) STAs are randomly distributed among groups; (ii) STAs are grouped based on respective MCS similitude (similar to [15,16]); and (iii) pure CSMA/CA-based contention (i.e., without RAW). Figure 21 and Figure 22 show throughput and fairness performance, respectively, for three different MCS random distribution approaches among the STAs composing the scenario: (i) MCSs are uniformly distributed, (ii) MCSs are distributed with 50% probability of being the slowest MCS (i.e., MCS 10), and the remaining 50% is assigned any MCS from MCS 0 to MCS 9 at random, following a uniform distribution, and (iii) MCSs are distributed with 50% probability of being the fastest MCS (i.e., MCS 9), and 50% probability of being any of the remaining MCSs (uniformly distributed). 

Results show that the GA-based grouping strategy provides a good trade-off between throughput and fairness performance in front of the other approaches. Obviously, when no grouping strategy is applied, fairness is maximized (15.98% above GA-based strategy, on average), but at the cost of minimizing overall throughput (41.04% below GA-based strategy, on average). The approach in which STAs are randomly distributed among groups does not show a good balance between throughput and fairness performances either (on average, fairness is 7.30% above GA-based strategy, throughput is 13.94% below GA-based strategy). On the other hand, the solution with STAs grouped based on their MCS similitude, results in unfair behavior for scenarios including a large amount of slow STAs (cf. Figure 22b) (on average, fairness is 8.54% below GA-based strategy).

## 5. Conclusions and Future Work

In this paper, we have proposed a grouping strategy method for IEEE 802.11ah networks based on the usage of a genetic algorithm. In the first place, we validate the proposal and tune the GA parameters to get the best fitness result in a reduced amount of time. We choose an initial population composed of *P* = 15 individuals (14 randomly generated, one preset according to previous experiences), and a maximum number of RAW groups of *R* = 12. As the computational time required for the convergence of the GA was high, we propose an alternative method for throughput calculation, thus, considerably reducing the complexity and the computational time of the algorithm (a reduction of around 10,000 times), while only a decrease of around 2% is observed in the fitness value. With the new fitness calculation, we provide a comparison of different alternatives with regard to parent selection, crossover and mutation methods. For parent selection, we choose the RBS method, as it presents a significant decrease in the computational time (around 20%). With respect to crossover, the single-point choice provides a further reduction of around 23%. For mutation, the RSM method is selected, employing a segment length of 5 and a mutation probability of 0.21, as it offers better fitness and computational time performance. We also use a more constrained device to run the GA, a Raspberry Pi 3B+, where the grouping method converges in around 5 s. Finally, we conclude the evaluation with a comparison of the GA-based grouping strategy with other grouping approaches, thus showing that the proposed mechanism provides a good trade-off between throughput and fairness performance.

As part of our future work, we plan to analyze additional mechanisms for reducing the computational time of the algorithm. We also aim to study the usage of adaptive mutation algorithms for the mutation phase, which we believe is an alternative to explore for obtaining improved fitness and computational time performance.

## Figures and Tables

**Table 1:** Alternatives for initial population.

Cases	Description
X RNDSTA FIX Y IND	X STAs with random MCS. Population of Y individuals, one is fixed.
X RNDSTA RND	X STAs with random MCS. Random population of 20 individuals.
X STA FIX Y IND	X STAs with X/11 STAs per MCS (MCS 0 to 11). Population of Y individuals, one is fixed.
X STA RND	X STAs with X/11 STAs per MCS (MCS 0 to 11). Random population of 20 individuals.

**Table 2:** Reduction of accuracy for the average fitness value of the best individual, and of all the individuals, using the new fitness function.

Cases	Diff. Best Individual (%)	Diff. All Individuals (%)
33 STA RND 20 IND	1.76	2.11
33 STA FIX 15 IND	1.17	1.20
33 RNDSTA RND 20 IND	2.15	2.33
33 RNDSTA FIX 15 IND	2.44	2.50
55 STA RND 20 IND	0.83	0.57
55 STA FIX 15 IND	0.32	0.39
55 RNDSTA FIX 15 IND	0.95	0.94

**Table 3:** Comparison for the computational time (s) between the original and the new fitness function.

Cases	Original (s)	New (s)
33 STA RND 20 IND	4050.445	0.684
33 STA FIX 15 IND	4666.563	0.409
33 RNDSTA RND 20 IND	4186.109	0.682
33 RNDSTA FIX 15 IND	2898.486	0.400
55 STA RND 20 IND	6288.776	1.009
55 STA FIX 15 IND	4666.563	0.616
55 RNDSTA FIX 15 IND	4353.809	0.640

**Table 4:** Computational time results using a Raspberry Pi 3B+.

Fitness	Crossover	Mutation	Time (s)
Original	Original	Original	70,261.848
New (Section 4.2)	Original	Original	7.737
New (Section 4.2)	Original	RSM	7.486
New (Section 4.2)	Single-Point	Original	5.621
New (Section 4.2)	Single-Point	RSM	5.145

## Data Availability

Not applicable.
